# Fried-Yennie Gauge in Pseudo-QED

**DOI:** 10.3390/e26020157

**Published:** 2024-02-11

**Authors:** Ana Mizher, Alfredo Raya, Khépani Raya

**Affiliations:** 1 Instituto de Física Teórica, Universidade Estadual Paulista, Rua Dr. Bento Teobaldo Ferraz, 271-Bloco II, São Paulo 01140-070, Brazil; 2 Laboratório de Física Teórica e Computacional, Universidade Cidade de São Paulo, R. Galvão Bueno, 868, Liberdade, São Paulo 01506-000, Brazil; 3 Centro de Ciencias Exactas, Universidad del Bío-Bío, Casilla 447, Chillán 3800708, Chile; raya@ifm.umich.mx; 4 Instituto de Física y Matemáticas, Universidad Michoacana de San Nicolás de Hidalgo, Morelia 58040, Michoacán, Mexico; 5 Departamento de Ciencias Integradas, Centro de Estudios Avanzados en Física, Matemática y Computacion, Facultad de Ciencias Experimentales, Universidad de Huelva, 21071 Huelva, Spain; khepani.raya@dci.uhu.es

**Keywords:** pseudo-QED, Fried-Yennie gauge, infrared behavior

## Abstract

The Fried-Yennie gauge is a covariant gauge for which the mass-shell renormalization procedure can be performed without introducing spurious infrared divergences to the theory. It is usually applied in calculations in regular Quantum Electrodynamics (QED), but it is particularly interesting when employed in the framework of pseudo-QED (PQED), where fermions are constrained to 2 + 1 dimensions while the dynamical fields interacting with these fermions live in the bulk of a 3 + 1 space. In this context, the gauge parameter can be adjusted to match the power of the external momentum in the denominator of the photon propagator, simplifying the infrared region without the need for a photon mass. In this work, we apply this machinery, for the first time, to PQED, generalizing the procedure to calculate the self energy in arbitrary dimensions, allowing, of course, for different dimensionalities of fermions and gauge fields.

## 1. Introduction

For a few decades, the field of condensed matter has provided physical realization of systems that can be associated with manifold others. more than the (topologically trivial) 3-space and 1-time dimensions ordinarily considered in high-energy physics. Single-layer materials, quantum wires and nanotubes are some examples of a plethora of possible arrangements that present alternative dimensionalities (see, for instance, [1]). Among these, systems arranged in 2-space dimensions have been a hot topic for several years [2]. The surface of liquid helium [3] or the interface of heterostructures are representatives of systems that harbor interesting properties deriving from their space structure. Also, 2D antiferromagnetic insulators [4] may give rise to a high $T_{c}$ superconductor [5,6,7,8,9] and, on the other hand, (2 + 1)D electron systems, when placed in a magnetic field, generate new phenomena in the realm of the Quantum Hall effect [10], such as the fractionalization of charge and statistics, statistical transmutation, and so on [11,12].

More recently, the discovery of graphene [13,14,15], along with a relatively simple method for its synthesis in the laboratory, have triggered new interest in (2 + 1)D systems. After the discovery, it was quickly shown that, among several remarkable mechanical and electrical properties, the charge carriers in graphene differ from most condensed matter systems [16], exhibiting a quasi-particle behavior that much resembles relativistic systems, despite having a Fermi velocity around 300 times lower than the speed of light. This feature is shared with a few of the aforementioned (2 + 1)D systems but theoretically demonstrated in a clear and simple way in honeycomb lattices. Given this relativistic-like character, it is not surprising that the continuous limit of the tight-binding approach usually applied to describe these systems yields the Dirac equation in (2 + 1)D. It was later shown that this framework is suitable for describing a series of other materials (planar or not) [2] and also the later discovered topological insulators [17,18,19]. Due to the common dispersion relation presented by these, they are classified as a family of materials dubbed Dirac materials.

In general, interaction with fields may affect the electronic properties of any material. It is therefore interesting to investigate the effects of interactions of this nature on relativistic-like planar materials. However, this kind of study presents a challenge, since the electrons are constrained to the plane while the dynamical fields are not. This was the main motivation behind the elaboration of pseudo-QED (PQED) [20]. Specifically, this framework, which is the focus of the present Special Issue, provides a mixed dimensional theory capable of describing fermionic systems in (2 + 1)D interacting with dynamical fields in (3 + 1)D, whether external or generated by the particles themselves. Although it is a non-local theory, it has been shown that it respects unitarity [21] and causality [22].

In fact, mixed dimensions are intrinsically present in other fields of physics, appearing in approaches like braneworld [23]. Inspired by this framework, a generalization of mixed dimensional theories was later developed called reduced-QED (RQED). This procedure proposes a treatment of systems of fermions living in generic dimensions different from the gauge fields, as long as the dimension of the former is smaller than the dimension of the latter [24]. RQED has also been widely applied for the particular case of planar materials interacting with dynamical fields. A comprehensive review on this subject, including PQED and RQED main results, can be found in [25].

The development of both approaches follows similar paths and consists of dimensionally reducing the gauge fields, defining an effective theory totally in (2 + 1)D that accounts for the projection of the gauge field on the plane. This procedure has been widely used to calculate chiral symmetry breaking in planar systems, mainly making use of Schwinger–Dyson techniques [26,27,28,29,30]. Renormalization in RQED was investigated in [31,32,33,34], and its scale invariance to all orders was proved in [35]. A renormalization group was also applied to investigate the gap in materials like diselenide (${\mathrm{WSe}}_{2}$) and molybdenumm disulfide (${\mathrm{MoS}}_{2}$) [36]. Aspects of the Chern–Simons theory, which is intimately related to PQED, were explored in [37,38,39,40]. Other aspects of the theory, like anisotropy in strained graphene [41], RQED in curved space [42], and supersymmetric PQED [43] were analyzed. The effects of a parity anomaly associated with a chemical potential were explored in [44].

In the present contribution, we will explore the implementation in PQED of a technique that makes the infrared sector of gauge theories more treatable and, although PQED is better behaved in the infrared compared to ordinary QED, it is still very useful for the regularization of this theory. It consists of performing dimensional regularization in the so-called Fried-Yennie gauge [45] and manipulating the expressions in a way that the mass-shell renormalization scheme can be implemented without introducing artificial infrared divergences.

This work is organized as follows. In Section 2, we introduce the Fried-Yennie gauge and calculate the self energy in arbritary dimensions, generalizing the formalism (initially applied to ordinary ${\mathrm{QED}}_{4}$). In Section 3, we apply the machinery developed in Section 2 to ordinary QED, checking that our approach reproduces the known results, and apply it to PQED. In Section 4, we summarize. Appendix A, Appendix B, Appendix C and Appendix D are dedicated to scrutinizing some of the calculations presented in the body of the manuscript.

## 2. The Fried-Yennie Gauge in D-Dimensions

### 2.1. Setting the Stage

The Fried-Yennie gauge has been explored in the context of quantum chromodynamics [46] to explore the quark self-energy with a gauge boson propagator of the following form:

(1) $$D_{\beta }^{\mu \nu }\left(k\right)=-{\left(\frac{\lambda_{d}}{k^{2}}\right)}^{\gamma_{d}}\left(g^{\mu \nu }+\beta \frac{k^{\mu }k^{\nu }}{k^{2}}\right)\;,$$

where β is the gauge parameter introduced to ensure transversality in x-space. In the context of mixed-dimensional theories, $\gamma_{d}$ depends on the space-time dimensionality in which fermions live. The quark propagator is expressed as usual,

(2) $$S\left(p\right)={\left[\gamma \cdot p-m-\Sigma \left(p\right)\right]}^{-1}\;,$$

but the self energy is expected to be cast as follows:

(3) $$\Sigma \left(p\right)=A+B\;\left(\gamma \cdot p-m\right)+C\left(p\right){\left(\gamma \cdot p-m\right)}^{2}\;.$$

This representation requires *A* to vanish since the self energy must vanish in the mass-shell (γ·p−m)=0, where *m* is the physical electron mass; also, as discussed in [47], *B* is connected to the electron wavefunction renormalization constant $Z_{2}$, via $Z_{2}=1/\left(1-B\right)$. Furthermore, in order for the expansion of Equation (3) to be well defined,

(4) $$\lim_{\gamma \cdot p\to m}\left[\left(\gamma \cdot p-m\right)C\left(p\right)\right]=0\;.$$

That said, the self-energy in Minkowsky space reads as follows:

(5) $$\begin{array}{ccc} -i\Sigma (p) & = & i\;\delta m+\int \frac{d^{f}k}{{\left(2\pi \right)}^{d}}\left[-ie\left(d\right)\gamma_{\mu }\right]\frac{i}{\gamma \cdot (p-k)-m}\\ & \times & \left[-ie\left(d\right)\gamma_{\nu }\right]\left[iD_{\beta }^{\mu \nu }\left(k\right)\right]\;. \end{array}$$

Here, $\delta_{m}=m-m_{0}$ acts as a counterterm ($m_{0}$ the bare quark mass) and $e\left(d\right)=\mu^{\epsilon }e$ is the electron charge (As usual, μ and ϵ are the regulators within a dimensional regularization scheme for *d*-dimensions). Here, *d* is the space-time dimension where the fermions live. For notation convenience, in the following, we shall employ Dirac notation γ·p→p. Thus, the self-energy reads as follows:

(6) Σ(p)=−δm+I,I=∫kγμ[(p−k)+m]γν(k2−2k·p+p2−m2)(k2)γdgμν+βkμkνk2.

where the integration symbol stands for

(7) $$\int_{k}:={\left(\lambda_{d}\right)}^{\gamma_{d}}\left(\frac{\alpha_{em}}{4\pi }\right){\left(4\pi \mu^{2}\right)}^{\epsilon }\int \frac{d^{d}k}{i\pi^{d/2}}\;.$$

Written in this way, the integral I in Equation (6) can be split as $I=I_{F}+\beta I_{\beta }$, such that

(8) IF=∫k(2−d)(p−k)+dm(k2−2k·p+p2−m2)(k2)γd,

(9) Iβ=∫k2k·pk−k2(p+k+m)(k2−2k·p+p2−m2)(k2)γd+1.

Focusing on the former, we introduce a Feynman parametrization to combine the denominators, obtaining

(10) IF=∫u∫k(2−d)(p−k)+dm(k2−2uk·p+u(p2−m2))γd+1,=Nγd+1(d)∫u[(2−d)(1−u)p+dm]1(M2)γD,

where the second line is derived using the formulas from Appendix A. We have adopted the following notation:

(11) $$\begin{array}{ccc} \int_{u} & := & \gamma_{d}\int_{0}^{1}du\;{\left(1-u\right)}^{\gamma_{d}-1} \end{array}$$

(12) $$\begin{array}{ccc} M^{2} & := & u(m^{2}-\left(1-u\right)p^{2}) \end{array}$$

(13) $$\begin{array}{ccc} \gamma_{D} & := & \gamma_{d}+1-d/2\;, \end{array}$$

(14) $$\begin{array}{ccc} N_{\alpha }^{\left(d\right)} & := & {\left(\lambda \right)}^{\gamma_{d}}\left(\frac{\alpha_{em}}{4\pi }\right)\frac{\Gamma (\alpha -d/2)}{\Gamma (\alpha )}{\left(-1\right)}^{\alpha }{\left(4\pi \mu^{2}\right)}^{\epsilon } \end{array}$$

A similar procedure is followed for the gauge term contribution, Equation (9), thus producing

(15) Iβ=γd+1γd∫u∫k2(1−u)k·pk(k2−2uk·p+u(p2−m2))γd+2,−∫u∫kp+k+m(k2−2uk·p+u(p2−m2))γd+1.

The second line can be evaluated just as in the $I_{F}$ case, while the first, as discussed in Appendix B, requires more attention. We thus have

(16) Iβ=1γdNγd+1(d)∫u[γdm−{(1−u)(1+γd−2γD)}p]1(M2)γD.

Notice that, if $d=d_{0}-2\epsilon$, where $d_{0}$ is the integer space-time dimension, then (as we shall see) $\gamma_{D}=\epsilon$. This result shall be used in the subsequent procedure. Collecting all the terms, one arrives at

(17) Σ(p)=−δm+Nγd+1(d)∫uf^mm+f^pp1(M2)ϵ,

where we have defined

(18) $$\begin{array}{ccc} {\hat{f}}_{m} & := & d+\beta \;,\\ {\hat{f}}_{p} & := & \left(1-u\right)\left(\left(2-d\right)-\frac{\beta }{\gamma_{d}}\left(1+\gamma_{d}-2\epsilon \right)\right) \end{array}$$

In the next subsection, we discuss how to match the above expression with the representation from Equation (3).

### 2.2. Identifying The Dressing Functions

First of all, the counterterm δm is fixed by the requirement that A=0, arising from the on-shell condition

(19) A=limp→mΣ(p),

which implies

(20) $$\begin{array}{ccc} \frac{\delta m}{m} & = & N_{\gamma_{d}+1}^{\left(d\right)}\int_{u}\left[{\hat{f}}_{m}+{\hat{f}}_{p}\right]\frac{1}{{\left(u^{2}m^{2}\right)}^{\epsilon }}\;\\ & = & {\bar{N}}_{\gamma_{d}}{\left(4\pi \frac{\mu^{2}}{m^{2}}\right)}^{\epsilon }\frac{\Gamma (1-2\epsilon )\Gamma (\epsilon )}{\Gamma (\gamma_{d}+2-2\epsilon )}\left(2\gamma_{d}+d\left(1-2\epsilon \right)\right)\;, \end{array}$$

independent of β, and where

(21) $${\bar{N}}_{\gamma_{d}}={\left(-1\right)}^{\gamma_{d}+1}\lambda_{d}^{\gamma_{d}}\left(\frac{\alpha_{em}}{4\pi }\right)\;.$$

Thus, Equation (17) can be rearranged as follows:

(22) Σ(p)=Nγd+1(d)∫u(f^m+f^p)m1(M2)ϵ−1(u2m2)ϵ+f^p(p−m)1(M2)ϵ.

As can be noted, the second line already displays a (p−m) factor, which will be useful to us when identifying *B*. Furthermore, as discussed in Ref. [47], the bracketed term in the first line can be recast so as to extract an overall (p−m) factor as well.

Let $M^{2}=um^{2}G$, with

(23) $$\begin{array}{ccc} G & := & u+\kappa (1-u)\;, \end{array}$$

(24) κ:=m2−p2m2=−1m2(p+m)(p−m).

Then, we can appeal to the following identity:

(25) $$\frac{1}{G^{\epsilon }}-\frac{1}{u^{\epsilon }}=-\epsilon \kappa \left(1-u\right)\int_{0}^{1}dv\;\frac{1}{{\bar{G}}^{1+\epsilon }}\;,$$

with $\bar{G}=u+\kappa \left(1-u\right)v$, such that, the combination of Equations (22) and (25) produces

(26) Σ(p)=Nγd+1(d)(p−m)∫u1(um2)ϵf^p1Gϵ+ϵ(f^m+f^p)(1−u)(p+m)m∫01dv1G¯1+ϵ.

Given the representation from Equation (3), and the fact that A=0, taking the limit p→m inside the integral yields the value of *B*:

(27) $$B=N_{\gamma_{d}+1}^{\left(d\right)}\int_{u}\frac{1}{{\left(um^{2}\right)}^{\epsilon }}\left\{{\hat{f}}_{p}\frac{1}{u^{\epsilon }}+2\epsilon \left({\hat{f}}_{m}+{\hat{f}}_{p}\right)\left(1-u\right)\int_{0}^{1}dv\frac{1}{u^{1+\epsilon }}\right\}\;.$$

Performing the evaluation of the integrals, one obtains

(28) $$\begin{array}{ccc} B & = & -{\bar{N}}_{\gamma_{d}}{\left(4\pi \frac{\mu^{2}}{m^{2}}\right)}^{\epsilon }\frac{\Gamma (1-2\epsilon )\Gamma (\epsilon )}{\Gamma (\gamma_{d}+2-2\epsilon )}\gamma_{d}\left(2\gamma_{d}+d\left(1-2\epsilon \right)\right)\\ & = & -\gamma_{d}\frac{\delta m}{m}\;, \end{array}$$

thus showing a direct relationship with the counterterm δm. Equations (20) and (28) generalize the QED result, presented in [47], for arbitrary dimensions. The substraction of the *B* contribution to Equation (26) enables us to identify

(29) (p−m)2C(p)=Nγd+1(d)(p−m)∫u1(um2)ϵ×f^p1Gϵ−1uϵ+ϵ(f^m+f^p)(1−u)×∫01dv(p+m)m1G¯1+ϵ−2u1+ϵ.

Once again, one needs to perform integration tricks in order to extract a global (p−m)2 factor. In the second line, the bracketed term can be expressed in a more useful way by employing the identity from Equation (25). The integrand of the last line, on the other hand, is split as follows:

(30) (p+m)m1G¯1+ϵ−2u1+ϵ→p−mm1G¯1+ϵ+21G¯1+ϵ−1u1+ϵ,

so that we have extracted (p−m) for the first term and one can use the following integral identity for the other piece:

(31) $$\int_{0}^{1}dv\left(\frac{1}{{\bar{G}}^{1+\epsilon }}-\frac{1}{u^{1+\epsilon }}\right)=-\left(1+\epsilon \right)\kappa \left(1-u\right)\int_{0}^{1}dv\frac{1-v}{{\bar{G}}^{2+\epsilon }}\;.$$

Therefore, one obtains

(32) C(p)=Nγd+1(d)1m∫u∫01dvϵ(um2)ϵ(1−u)×1G¯1+ϵf^pp+mm+(f^p+f^m)+2(1−v)G¯2+ϵf^p+f^m(1+ϵ)(1−u)p+mm.

Before discussing the case of PQED, we shall comment on some issues about C(p), related to the convergence of the integral, as well as the selection process of β and $\gamma_{d}$ for a given space-time dimensionality of the fermion.

### 2.3. Scrutinizing C(p): Convergence and Gauge Fixing

Before going to the particular case of PQED, let us further explore C(p). For analysis, it turns out to be convenient to rewrite Equation (32) (here, ${\hat{f}}_{p}:=\left(1-u\right){\tilde{f}}_{p}$):

(33) C(p)=Nγd+1(d)1m∫u∫01dvϵ(um2)ϵ(1−u){pmuf˜pG¯1+ϵ+p+mm(1−2u)1G¯1+ϵ−2u(1+ϵ)(1−u)1−vG¯2+ϵf˜p+1G¯1+ϵ+2p+mm(1+ϵ)(1−u)1−vG¯2+ϵ(f^m+f˜p)}.

It is argued that the last line of the above expression is not well behaved at small values of ϵ, Ref. [47]. For this reason, and for the simplicity it entails, it is convenient to fix the value of β from the requirement that

(34) $${\hat{f}}_{m}+{\tilde{f}}_{p}=\frac{2\gamma_{d}-\beta \left(1-2\epsilon \right)}{\gamma_{d}}=0\;,$$

This constraint leads to a link between β and $\gamma_{d}$,

(35) $$\beta \left(\gamma_{d}\right)=\frac{2\gamma_{d}}{1-2\epsilon }\;.$$

which is a particular gauge-fixing for the arbitrary power-like behavior for the photon propagator, in which the transversality condition dictates the following [46]:

(36) $$\beta \left(\gamma_{d}\right)=\frac{2\gamma_{d}}{d-1-2\gamma_{d}}\;;$$

in our case, assuming the photon lives in a 4-dimensional space-time [31] (and recalling that $d=d_{0}-2\epsilon$),

(37) $$\gamma_{d}=\frac{d_{0}-2}{2}\;,$$

also confirming Equation (13), namely $\gamma_{D}=\epsilon$. For the above, in QED, we have $\gamma_{d}$, such that we recover the well-known result β=2/(1−2ϵ). Also in QED, the second line of Equation (33) vanishes, such that C(p) is completely determined by the p term from the first line. This is not the general case where, given Equations (35) and (37), one is left with

(38) C(p)=Nγd+1(d)f˜pm2∫u∫01dvϵ(1−u)(um2)ϵ{pu1G¯1+ϵ+(p+m)(1−2u)1G¯1+ϵ−2u(1+ϵ)(1−u)(1−v)G¯2+ϵ}.

So, we have properly identified each part of the self-energy: the counterterm (Equation (20)), the *B* constant (Equation (28)) and the C(p) dressing function (Equation (38)). We now turn our attention to the PQED case.

## 3. The Fried-Yennie Gauge in PQED

Let us recall that, in PQED, $d_{0}=3$ is implied and so $\gamma_{d}=1/2$, λ=1/4 and β=1/(1−2ϵ). Therefore, one has

(39) $$\begin{array}{ccc} \frac{\delta m}{m} & = & {\bar{N}}_{1/2}{\left(4\pi \frac{\mu^{2}}{m^{2}}\right)}^{\epsilon }\frac{\Gamma (1-2\epsilon )\Gamma (\epsilon )}{\Gamma (5/2-2\epsilon )}4{\left(1-\epsilon \right)}^{2}\;\\ & \overset{\epsilon \to 0}{\approx } & {\bar{N}}_{1/2}\frac{16}{3\sqrt{\pi }}(\frac{1}{\epsilon }+[\ln\left(4\pi \frac{\mu^{2}}{m^{2}}\right)\\ & + & 2\frac{\Gamma^{\prime }\left(5/2\right)}{\Gamma (5/2)}-2+\gamma_{E}])+O\left(\epsilon \right)\;, \end{array}$$

with $\gamma_{E}$ being the Euler gamma function. According to Equation (28), *B* is merely obtained by multiplying the above expression by $-\gamma_{d}=-1/2$.

Concerning C(p), it is convenient to separate the p and *m* contributions in Equation (38) as follows:

(40) C(p)=Cp(p)p+Cm(p)m=[Cp0(p)+Cm(p)]p+Cm(p)m,

where, again, $C_{m}\left(p\right)$ vanishes in ${\mathrm{QED}}_{4}$. The corresponding integrals are convergent and the limit ϵ→0 can be taken safely (see Appendix D). The final result for the coefficient $C_{p_{0}}$ in ${\mathrm{QED}}_{4}$ is

(41) $$C_{p_{0}}^{QED_{4}}\left(p\right)=\frac{3{\bar{N}}_{1}}{m^{2}{\left(\kappa -1\right)}^{2}}\left(-1+\kappa \left(1-\log\kappa \right)\right).$$

Now, focusing on the PQED case, where d=3−2ϵ and $\gamma_{d}=1/2$, we find

(42) Cp0PQED=−16N¯1/23m2κπ53+Logκ4−F1(0,1,0,0)22,0,52,κ−1κ.

and

(43) Cm=−16N¯3m2κπ(−2+2−κ2(H3/2+Log[κ])−32(2+κ)F1(0,1,0,0)21,0,32,κ−1κF1(0,1,0,0)21,0,52,κ−1κ+(2κ)F1(0,1,0,0)22,0,52,κ−1κ)

The full self-energy for PQED in the Fried-Yennie gauge is given by Equation (3), where the coefficients can be obtained from Equations (39), (42) and (43). Note that the coefficient *B* depends on the mass through $\mathrm{Log}(\mu^{2}/m^{2})$. After mass renormalization, one can insert this coefficient into the expression for the self-energy and check that the infrared limit yields to a dependence of $m\mathrm{Log}(\mu^{2}/m^{2})$ for the second term in Equation (3). This clearly vanishes in the limit m→0. On the other hand, one can easily check that $C_{p_{0}}^{PQED}$ and $C_{m}$ go with $1/m^{2}$. The integrals in Equation (38) are not *m*-dependent in the infrared and the only dependence is on the overall $1/m^{2}$ factor in this equation. When inserted in Equation (3), the dependence on the mass in the third term is lifted in the infrared regime and gives a finite contribution for the self-energy. Under these considerations, it is clear that the self-energy is well-defined in the infrared for any value of the mass, including the limit of vanishing mass.

## 4. Summary

In this article, we have carried out an explicit one-loop calculation of the fermion self-energy in the mixed dimensional theory of Reduced or Pseudo-QED with 4-dimensional photons and 3-dimensional fermions. We have selected to work with the covariant Fried-Yennie gauge and implemented an explicit mass-shell renormalization of Σ(p), which acquires the form shown in Equation (3). The present calculation generalizes the previously known case of ${\mathrm{QED}}_{4}$ carried out in [47], motivated by the Coulomb static interaction among charge carriers in low-energy graphene. Although one usually considers graphene as a gapless system, there are a number of proposals for mechanisms which can open the gap and thus induce a mass for electrons in the material, including self interactions [24,25,26,27,28,29,30]. In this particular context, our approach is suitable for calculations.

The fermion self energy in arbitrary dimensions is defined by the functions in Equations (28) and (38). For the particular case of PQED, these expressions simplify to the ones shown in Equations (39), (42) and (43) once dimensionally regularized. It should be noticed that these expressions do not introduce any spurious infrared divergences whatsoever. Of course, the limit m→0 is straightforward to obtain and, as a result, the self energy in Equation (3) is finite, as mB(p) and $m^{2}C\left(p\right)$ are finite as p→0. This is a nice feature of the Fried-Yenni gauge.

Finally, it is worth mentioning that the gauge adopted here is considered to calculate the structure of the fermion–photon vertex correction to this theory and, hence, the anomalous magnetic moment of charge carriers in graphene. On the other hand, the massive fermion propagator in an arbitrary gauge is also considered. The results will be reported elsewhere.

## Data Availability

No new data were created or analyzed in this study. Data sharing is not applicable to this article.

## Appendix A. About the 4-Momentum Integrals

Recalling that

(A1) $$\int_{k}:={\left(\lambda \right)}^{\gamma_{d}}\left(\frac{\alpha_{em}}{4\pi }\right){\left(4\pi \mu^{2}\right)}^{\epsilon }\int \frac{d^{d}k}{i\pi^{d/2}}\;,$$

the following useful formulas have been employed throughout this work:

(A2) $$I_{\alpha }^{S}\left(d\right):=\int_{k}\frac{1}{{\left(k^{2}+2k\cdot v-z\right)}^{\alpha }}=N_{\alpha }^{\left(d\right)}\frac{1}{{\left(v^{2}+z\right)}^{\alpha -d/2}}\;,$$

(A3) $$I_{\mu ,\alpha }^{V}\left(d\right):=\int_{k}\frac{k_{\mu }}{{\left(k^{2}+2k\cdot v-z\right)}^{\alpha }}=-v_{\mu }I^{S}\left(d\right)\;,$$

(A4) $$\begin{array}{ccc} I_{\mu \nu ,\alpha }^{T}\left(d\right) & := & \int_{k}\frac{k_{\mu }k_{\nu }}{{\left(k^{2}+2k\cdot v-z\right)}^{\alpha }}\\ & = & I_{\alpha }^{S}\left(d\right)\left(v_{\mu }v_{\nu }-\frac{1}{2}g_{\mu \nu }\frac{v^{2}+z}{\alpha -d/2-1}\right)\;. \end{array}$$

Here,

(A5) $$N_{\alpha }^{\left(d\right)}:={\left(\lambda \right)}^{\gamma_{d}}\left(\frac{\alpha_{em}}{4\pi }\right)\frac{\Gamma (\alpha -d/2)}{\Gamma (\alpha )}{\left(-1\right)}^{\alpha }{\left(4\pi \mu^{2}\right)}^{\epsilon }$$

## Appendix B. On the *β* Integral

Let us consider the gauge term contribution to the self energy, written down in Equation (9):

(A6) Iβ=γd+1γd∫u∫k2(1−u)k·pk(k2−2uk·p+u(p2−m2))γd+2,−∫u∫kp+k+m(k2−2uk·p+u(p2−m2))γd+1.

The evaluation of the second line is straightforward. For the first line, let us first consider the momentum integration:

∫k2k·pk(k2−2uk·p+u(p2−m2))γd+2=2pμγν∫kkμkν(k2−2uk·p+u(p2−m2))γd+2=2pμγνu2pμpν−12M2γd+2−1−d/2gμνIγd+2S(d)=−2pu2p2−12M2γDγDγd+11M2Iγd+1S(d)=2p12−u2p2M2γD1γd+1Nγd+1(d)1(M2)γD,

where we have employed the formula from Equation (A4). Therefore, we have

(A7) Iβ=2γdNγd+1(d)∫u(1−u)12−γDu2p2M2p1(M2)γD−Nγd+1(d)∫u[(1+u)p+m]1(M2)γD;

or, more conveniently,

(A8) Iβ=1γdNγd+1(d)∫u[{(1−u)−γd(1+u)}p−γdm]1(M2)γD−2γdNγd+1(d)∫u(1−u)γDu2p2p1(M2)γD+1.

The second line above can be recast, using integration by parts, in order to have the same power of $M^{2}$, thus producing

(A9) $$\int_{u}\left(1-u\right)\gamma_{D}u^{2}p^{2}\frac{1}{{\left(M^{2}\right)}^{\gamma_{D}+1}}=\int_{u}\left[\left(1-\gamma_{D}\right)-\left(1+\gamma_{d}-\gamma_{D}\right)u\right]\frac{1}{{\left(M^{2}\right)}^{\gamma_{D}}}\;,$$

which holds for $\gamma_{D}<0$ and $\gamma_{d}>0$. Finally, we arrive at a rather compact expression for $I_{\beta }$:

(A10) Iβ=1γdNγd+1(d)∫u[γdm−{(1−u)(1+γd−2γD)}p]1(M2)γD.

## Appendix C. More about *C*(*p*)

After performing the integral over dv, we can identify the coefficients accompanying p and *m* contributions to C(p):

(A11) C(p)=Cp(p)p+Cm(p)m=[Cp0(p)+Cm(p)]p+Cm(p)m,

where

(A12) $$C_{p_{0}}\left(p\right)=-{\bar{N}}_{\gamma_{d}}{\left(4\pi \frac{\mu^{2}}{m^{2}}\right)}^{\epsilon }\frac{\Gamma (\epsilon )}{\Gamma (\gamma_{d})}\frac{{\tilde{f}}_{p}}{\kappa }\int_{u}\frac{u}{{\left(um^{2}\right)}^{\epsilon }}\left(\frac{1}{G^{\epsilon }}-\frac{1}{u^{\epsilon }}\right)\;$$

and with the $C_{m}\left(p\right)$ dressing function, which vanishes in regular QED, being

(A13) $$\begin{array}{ccc} C_{m}\left(p\right) & = & {\bar{N}}_{\gamma_{d}}{\left(4\pi \frac{\mu^{2}}{m^{2}}\right)}^{\epsilon }\frac{\Gamma (1+\epsilon )}{\Gamma (\gamma_{d})}\frac{{\tilde{f}}_{p}}{\kappa }\int_{u}\frac{\left(1-u\right)}{{\left(um^{2}\right)}^{\epsilon }}\\ & \times & \left(2u\left[\frac{1}{G^{1+\epsilon }}-\frac{1}{u^{1+\epsilon }}\right]-\frac{\left(1-2u\right)}{(1-u)\epsilon }\left[\frac{1}{G^{\epsilon }}-\frac{1}{u^{\epsilon }}\right]\right)\;. \end{array}$$

## Appendix D. *C*(*p*) Integrals

The result of the first integral in Equation (38), that gives the coefficient of p alone, identified as $C_{p_{0}}$, is given by

(A14) ∫u∫01dv(1−u)γd(um2)ϵuG¯1+ϵ=(m2)−eκΓ[2−2ϵ]Γ[ϵ]Γ[2+γd−2ϵ]+κ−ϵπCsc[πϵ]F˜12[2−ϵ,ϵ,2+γd−ϵ,κ−1κ]Γ[ϵ−1],

where F12[a,b,c,d] is the regularized Hypergeometric function and Γ[x] is the Gamma function. The limit ϵ→0 can be taken safely, yielding

J1=Limitϵ→0f˜p4πμ2m2ϵ∫u∫01dv(1−u)γd(um2)ϵuG¯1+ϵ=−(d+2γd)κΓ[2+γd](−1+H1+γd+Log[κ]+F1(0,0,1,0)22,0,2+γd,κ−1κ−F1(0,1,0,0)22,0,2+γd,κ−1κ+F1(1,0,0,0)22,0,2+γd,κ−1κ).

Here, F1(i,j,k,l)2[a,b,c,d] (without the tilde) gives the derivative of order i,j,k,l of the Hypergeometric function with respect to variables a,b,c,d. We have also used the gauge condition $\beta =2\gamma_{d}/\left(1-2\epsilon \right)$ to obtain the expression above. For real values of the arguments κ and $\gamma_{d}$, the following derivatives of the Hypergeometric function vanish:

(A15) F1(0,0,1,0)22,0,2+γd,κ−1κ=0F1(1,0,0,0)22,0,2+γd,κ−1κ=0

such that the result can be simplified to

J1=−(d+2γd)κΓ[2+γd]−1+H1+γd+Log[κ]−F1(0,1,0,0)22,0,2+γd,κ−1κ.

In the case of QED, $\gamma_{d}=1$ and d=4 in the limit ϵ→0. Also, the following identity holds:

(A16) F1(0,1,0,0)22,0,3,κ−1κ=12(κ−1)21−4κ+3κ2+(2−4κ)Log[κ−1κ].

Considering this, the complete expression for $C_{p_{0}}^{QED_{4}}$, including all the coefficients from Equation (38), becomes

(A17) $$\begin{array}{ccc} C_{p_{0}}^{QED_{4}} & = & \frac{{\bar{N}}_{1}}{m^{2}}J_{1} \end{array}$$

(A18) $$\begin{array}{ccc} & = & \frac{3{\bar{N}}_{1}}{m^{2}\kappa {\left(\kappa -1\right)}^{2}}\left(\kappa^{2}-\kappa -\kappa^{2}\mathrm{Log}\left[\kappa \right]\right). \end{array}$$

For PQED, $\gamma_{d}=1/2$ and d=3−2ϵ, and the final expression, following the same steps used to obtain (A18), is

(A19) Cp0PQED=−16N¯1/23m2κπ53+Logκ4−F1(0,1,0,0)22,0,52,κ−1κ.

In the equation above, we have benefited from the relation $-1+H_{3/2}=5/3-\mathrm{Log}\left[4\right]$. The sum of the other two integrals in Equation (38), which gives the coefficient of p+m, identified as $C_{m}$, is given by the following (considering that other derivatives of the Hypergeometric functions that appear in the results vanish for real values of the parameters):

(A20) Limitϵ→0∫u∫01dv(1−2u)1G¯1+ϵ−2u(1+ϵ)(1−u)1G¯2+ϵ=γdκ2Γ[2+γd](−2+κ(1−2γd)+(2+κ(γd−1))(H1+γd+Log[κ])−(2+κ)(1+γd)F1(0,1,0,0)21,0,1+γd,κ−1κ+(2γd)F1(0,1,0,0)21,0,2+γd,κ−1κ+(2κ)F1(0,1,0,0)22,0,2+γd,κ−1κ).

For ${\mathrm{QED}}_{4}$, the coefficient $C_{m}$ associated with the integral above vanishes. This can be seen when applying the conditions $\gamma_{d}=1/2$, d=3−2ϵ and using the relations

(A21) F1(0,1,0,0)21,x,2,κ−1κ=κ−1−Log[κ]κ−1

(A22) F1(0,1,0,0)21,x,3,κ−1κ=3+κ(κ−4)+2Log[κ]2(κ−1)2

(A23) F1(0,1,0,0)22,x,3,κ−1κ=1+κ(3κ−4)+2Log[κ]−4κLog[κ]2(κ−1)2.

For PQED, the coefficient $C_{m}$ becomes

(A24) Cm=−16N¯3m2κπ(−2+2−κ2H3/2+Log[κ]−32(2+κ)F1(0,1,0,0)21,0,32,κ−1κF1(0,1,0,0)21,0,52,κ−1κ+(2κ)F1(0,1,0,0)22,0,52,κ−1κ).

## References

[1] Hoffmann R., Kabanov A., Artyom A., Golov A., Andrey A., Proserpio D.M. (2016). Homo Citans and Carbon Allotropes: For an Ethics of Citation. Angew. Chem. Int. Ed..

[2] Mounet N., Gibertini M., Schwaller P., Campi D., Merkys A., Marrazzo A., Sohier T., Castelli I.E., Cepellotti A., Pizzi G. (2018). Two-dimensional materials from high-throughput computational exfoliation of experimentally known compounds. Nat. Nanotechnol..

[3] Volovik G.E. (2009). The Universe in a Helium Droplet.

[4] Lado J.L., Sigrist M. (2018). Two-Dimensional Topological Superconductivity with Antiferromagnetic Insulators. Phys. Rev. Lett..

[5] Dorey N., Mavromatos N.E. (1992). QED3 and two-dimensional superconductivity without parity violation. Nucl. Phys. B.

[6] Farakos K., Mavromatos N.E. (1998). Dynamical gauge symmetry breaking and superconductivity in three-dimensional systems. Mod. Phys. Lett. A.

[7] Franz M., Tesanovic Z. (2001). Algebraic Fermi Liquid from Phase Fluctuations: “Topological” Fermions, Vortex “Berryons”, and QED_3_ Theory of Cuprate Superconductors. Phys. Rev. Lett..

[8] Herbut I.F. (2002). QED_3_ theory of underdoped high-temperature superconductors. Phys. Rev. B.

[9] Franz M., Tesanovic Z., Vafek O. (2002). QED_3_ theory of pairing pseudogap in cuprates: From *d*-wave superconductor to antiferromagnet via an algebraic Fermi liquid. Phys. Rev. B.

[10] von Klitzing K., Chakraborty T., Kim P., Madhavan V., Dai X., McIver J., Tokura Y., Savary L., Smirnova D., Rey A.M. (2020). 40 years of the quantum Hall effect. Nat. Rev. Phys..

[11] Prange R.E., Girvin S.M. (1989). “The Quantum Hall Effect” Graduate Texts in Contemporary Physics.

[12] Ezawa Z.F. (2013). Quantum Hall Effects: Recent Theoretical and Experimental Developments.

[13] Novoselov K.S., Geim A.K., Morozov S.V., Jiang D., Zhang Y., Dubonos S.V., Grigorieva I.V., Firsov A.A. (2004). Electric field effect in atomically thin carbon films. Science.

[14] Zhang Y., Tan Y.-W., Horst L.S., Kim P. (2005). Experimental observation of the quantum Hall effect and Berry’s phase in graphene. Nature.

[15] Novoselov K.S., Geim A.K., Morozov S.V., Jiang D., Katsnelson M.I., Grigorieva I.V., Dubonos S.V., Firsov A.A. (2005). Two-dimensional gas of massless Dirac fermions in graphene. Nature.

[16] Geim A., Novoselov K. (2007). The rise of graphene. Nat. Mater..

[17] Hasan M.Z., Kane C.L. (2010). Colloquium: Topological insulators. Rev. Mod. Phys..

[18] Qi X.-L., Zhang S.-C. (2011). Topological insulators and superconductors. Rev. Mod. Phys..

[19] Shankar R. (2018). Topological Insulators—A review. arXiv.

[20] Marino E. (1993). Quantum electrodynamics of particles on a plane and the Chern-Simons theory. Nucl. Phys. B.

[21] Marino E.C., Nascimento L.O., Alves V., Smith C.M. (2014). Unitarity of theories containing fractional powers of the d’Alembertian operator. Phys. Rev. D.

[22] Amaral R.L.P.G.d., Marino E.C. (1992). Canonical quantization of theories containing fractional powers of the d’Alembertian operator. J. Phys. A.

[23] Rubakov V.A. (2001). Large and infinite extra dimensions: An Introduction. Phys. Usp..

[24] Gorbar E.V., Gusynin V.P., Miransky V.A. (2001). Dynamical chiral symmetry breaking on a brane in reduced QED. Phys. Rev. D.

[25] Olivares J.A.C., Mizher A.J., Raya A. (2022). Non-perturbative field theoretical aspects of graphene and related systems. Rev. Mex. Fis..

[26] Báez-Cuevas J., Raya A., Rojas J.C. (2020). Chiral symmetry restoration in reduced QED at finite temperature in the supercritical coupling regime. Phys. Rev. D.

[27] Kotikov A., Teber S. (2016). Critical behaviour of reduced QED_4,3_ and dynamical fermion gap generation in graphene. Phys. Rev. D.

[28] Nascimento L.O., Alves V., Peña F.P., Smith C.M., Marino E. (2015). Chiral-symmetry breaking in pseudoquantum electrodynamics at finite temperature. Phys. Rev. D.

[29] Alves V., Elias W.S., Nascimento L.O., Jurićixcx V., Peña F. (2013). Chiral symmetry breaking in the pseudo-quantum electrodynamics. Phys. Rev. D.

[30] Albino L., Bashir A., Mizher A.J., Raya A. (2022). Electron-photon vertex and dynamical chiral symmetry breaking in reduced QED: An advanced study of gauge invariance. Phys. Rev. D.

[31] Teber S. (2012). Electromagnetic current correlations in reduced quantum electrodynamics. Phys. Rev. D.

[32] Kotikov A.V., Teber S. (2014). Two-loop fermion self-energy in reduced quantum electrodynamics and application to the ultrarelativistic limit of graphene. Phys. Rev. D.

[33] Teber S. (2014). Two-loop fermion self-energy and propagator in reduced QED_3,2_. Phys. Rev. D.

[34] Teber S., Kotikov A. (2018). Field theoretic renormalization study of reduced quantum electrodynamics and applications to the ultrarelativistic limit of Dirac liquids. Phys. Rev. D.

[35] Dudal D., Mizher A.J., Pais P. (2019). Exact quantum scale invariance of three-dimensional reduced QED theories. Phys. Rev. D.

[36] Fernández L., Alves V., Nascimento L.O., Peña F., Gomes M., Marino E.C. (2020). Renormalization of the band gap in 2D materials through the competition between electromagnetic and four-fermion interactions in large N expansion. Phys. Rev. D.

[37] Carrington M.E. (2019). Effect of a Chern-Simons term on dynamical gap generation in graphene. Phys. Rev. B.

[38] Olivares J.A.C., Albino L., Mizher A.J., Raya A. (2020). Influence of a Chern-Simons term in the dynamical fermion masses in reduced or pseudo QED. Phys. Rev. D.

[39] Magalhães G.C., Alves V., Marino E.C., Nascimento L.O. (2020). Pseudo Quantum Electrodynamics and Chern-Simons theory Coupled to Two-dimensional Electrons. Phys. Rev. D.

[40] Dudal D., Mizher A.J., Pais P. (2018). Remarks on the Chern-Simons photon term in the QED description of graphene. Phys. Rev. D.

[41] Carrington M.E., Frey A.R., Meggison B.A. (2020). Effect of anisotropy on phase transitions in graphene. Phys. Rev. B.

[42] Caneda P.I.C., Menezes G. (2021). Reduced quantum electrodynamics in curved space. Phys. Rev. D.

[43] Alves V., Gomes M., Petrov A.Y., da Silva A.J. (2023). On the supersymmetric pseudo-QED. Phys. Lett. B.

[44] Dudal D., Matusalem F., Mizher A.J., Rocha A.R., Villavicencio C. (2022). Half-integer anomalous currents in 2D materials from a QFT viewpoint. Sci. Rep..

[45] Fried H.M., Yennie D.R. (1958). New Techniques in the Lamb Shift Calculation. Phys. Rev..

[46] Boos E.E., Davydychev A.I. Infrared problems of describing the glueball and an estimation of its mass. Preprint INP MSU 88-21/42, Moscow, 1988.

[47] Adkins G.S. (1993). Fried-Yennie gauge in dimensionally regularized QED. Phys. Rev. D.

